# Does running at an angle affect running economy? Metabolic, kinematic, and EMG adaptations to running on a camber

**DOI:** 10.1007/s00421-025-06034-3

**Published:** 2025-10-29

**Authors:** Jong Min Park, Jane E. Nakamura, Adam D. Goodworth, Timothy A. VanHaitsma

**Affiliations:** 1 Department of Kinesiology, Westmont College, Santa Barbara, CA USA; 2 Department of Engineering, Westmont College, Santa Barbara, CA USA

**Keywords:** Camber, Cross-slope, Running economy, Kinematics, Electromyography, Balance

## Abstract

**Objectives:**

Distance runners will often encounter a camber during running. Camber refers to when the road is slanted on a cross-slope angle to promote water drainage. Camber can alter running mechanics and muscle activation patterns, which may affect running economy and cause injuries due to asymmetries in loading. This study investigates the effects of road camber on running economy and its interactions with running mechanics and muscle activation patterns.

**Methods:**

Twelve trained runners performed three 5 min treadmill runs at camber conditions of 0 deg, 3 deg, and 6 deg just below ventilatory threshold pace. Metabolic, kinematic, and EMG data were measured during the final two minutes of each trial.

**Results:**

Increasing camber caused significant changes in knee and trunk movement patterns and gastrocnemius muscle activation. However, there were no significant differences in metabolic measures.

**Conclusions:**

In experienced runners, the body may utilize a ground-up approach to maintain balance on a camber, with alterations that are not large enough to cause metabolic perturbations. This suggests that runners looking for their best performances do not need to avoid the cambered section of a road.

**Supplementary Information:**

The online version contains supplementary material available at 10.1007/s00421-025-06034-3.

## Introduction

Running has become an increasingly popular sport, with some of the most popular marathons being held largely on roads meant for automobiles. These roads are often built with a slight cross-slope, or camber, to allow for water to drain to the sides. Most cambers range from 3 degrees with road shoulder cambers increasing to 5 degrees or more (California S of. Highway Design Manual (HDM) 2020). Most runners will find themselves running on the side of the road for extended periods of time. However, running on these cambered surfaces often forces one foot to repeatedly land higher than the other, which may alter overall running mechanics and in turn induce performance decrements. Cambered surfaces have been postulated to introduce an environmental leg length inequality where runners are forced to compensate for these simulated leg-length differences with kinematic changes in the lower body (Damavandi et al. 2016; Mccaw 1992; White et al. 2004).

Previous literature has investigated the effects of banked curves on running kinematics, which expresses some similarities with running on cambered surfaces due to the need for frontal plane adaptations (Garie 2006; Gow 2005; Quintana 2020). However, previous research investigating kinematic changes with cambered running is relatively limited. One study found kinematic differences in the knee joint during cambered running, and hypothesized this change helped attenuate the force during lateral inclination (Gehlsen et al. 1989). Greater knee flexion has been observed in the elevated (or upslope) leg while running at 2.5 deg and 6 deg, respectively (Elmer and Asbill 2023; Sussman et al. 2001). On a 10 degree camber, changes in the hip, knee, and ankle were seen during the stance phase, where the elevated hip was more adducted and internally rotated while the elevated knee and ankle were more externally rotated (Damavandi et al. 2016). There were also differences (also at 10 deg) in intra-foot adaptations with increasing camber (Dixon et al. 2011). Overall, these kinematic adaptations of the foot, ankle, knee, and hip seem to be aimed at reversing the leg-length differences induced by camber.

The literature has also shown significant changes in gait characteristics induced by cambered running, which are often concurrent with kinetic changes (Damavandi et al. 2016). Timing differences in gait patterns have been observed with increasing camber (Damavandi et al. 2016), which are supported by findings on reductions in flight time, increases in ground contact time, and increases in duty factor on the elevated leg with increasing camber (Elmer and Asbill 2023). These changes in gait characteristics are accompanied by asymmetrical loading patterns, characterized by changes in mediolateral ground reaction forces to stabilize the body and minimize the risk of falling. Running on cambers of 3 deg and 6 deg shifted the point of force application in the feet (Willwacher et al. 2013), which is concurrent with changes in foot position and orientation during the stance phase (Dixon et al. 2011). Frontal plane changes place unique demands on the body’s balance control system, which may explain the previous literature that connects these changes in running kinematics and kinetics to changes in muscle recruitment patterns (Goodworth et al. 2013). However, previous research investigating EMG changes with camber are limited and contradictory. Significant differences in the activation of the tibialis anterior, gastrocnemius, and quadriceps were found during the stance phase while running on a camber of 5–7 deg (Unfried et al. 2013), while no significant differences in the aforementioned muscle groups were found at 6 deg in another study (Elmer and Asbill 2023).

Previous research investigating how each of these kinematic and EMG adaptations influence running economy is still new. Running economy is typically defined as the oxygen cost of running at a given submaximal velocity and is shown to be affected by a number of metabolic, kinematic, and EMG factors (Barnes and Kilding 2015; Chang and Kram 1999; Fletcher et al. 2009; Folland et al. 2017; Hoogkamer et al. 2016; Saunders et al. 2004; Shaw et al. 2014). Although running economy as a measure of running performance in highly trained distance runners is extensively backed by the literature (Hoogkamer et al. 2016; Saunders et al. 2004; Conley and Krahenbuhl 1980), the effects of kinematic and EMG changes on running economy have only been examined separately and on flat surfaces. The first study to explore these factors simultaneously did not control for shoe type beyond restricting the use of shoes with an embedded carbon plate, and did not individualize running speed according to each participant’s ventilatory threshold (Elmer and Asbill 2023). The current study serves to match each individual participant’s running economy with a steady-state pace slightly below their ventilatory threshold as deviations from such intensities have been shown to alter running economy (Fletcher et al. 2009). The purpose of this study was to investigate the effects of typical camber conditions on running economy at an individualized pace by analyzing its concurrent metabolic, kinematic, and EMG adaptations. It was hypothesized that running on a camber would produce significant changes in running kinematics and EMG but not significantly alter running economy in trained runners.

## Methods

### Participants

All experimental procedures in this investigation were reviewed and approved by the Westmont College Institutional Review Board (IRB) prior to beginning this study. The protocols and procedures were explained, and all participants provided written informed consent prior to testing. Participants (*N* = 12) consisted of recreationally trained individuals or collegiate cross-country athletes recruited by word of mouth (males = 12, mean ± SD; age = 29.5 ± 13.3 years, height = 179.6 ± 5.2 cm, weight = 71.2 ± 8.3 kg, VO_2max_ = 61.3 ± 7.8 mL/kg/min, training volume = 48.3 ± 27.7 km/week in the last 4 weeks). Three additional participants began the study but were excluded from the analysis due to sensor interference, sensors falling off, or running at a pace that had had anaerobic contribution due to having a respiratory exchange ratio (RER) exceeding 1.0 during one of the trials.

### Equipment and Protocol

#### Protocol overview and exercise protocols

The study consisted of a within-subjects repeated measures test design in which participants completed two separate protocols in a single visit: an initial graded exercise test (GXT) and three 5 min runs (order randomized) with 5 min rest breaks in between. A single-visit test design was employed to reduce the possibility of intersession variability. Between the two protocols, participants were instructed to rest for 15 min to allow for adequate recovery following a maximal exercise bout. Previous literature has shown that running economy is sensitive to shoe type, especially to those with an embedded carbon plate (Chen et al. 2014; Healey and Hoogkamer 2022; Hoogkamer et al. 2018; Joubert and Jones 2022; Oh and Park 2017; Oleson et al. 2005; Roy and Stefanyshyn 2006; Stefanyshyn and Nigg 1997; Worobets et al. 2014). Thus, participants were instructed to run in a pair of Brooks Ghost 15 shoes, which do not have an embedded carbon plate, to control for any possible variability due to shoe type. Two pairs of the Brooks Ghost 15 were available in sizes men’s US 10.5 and men’s US 9 thereby reducing the current study’s sample size to individuals who were within a half-size of the available shoe sizes. Prior to each protocol, participants were instructed to perform a light warm-up lasting approximately 5 min.

#### Graded exercise test protocol

The purpose of the graded exercise test (GXT) was to estimate the speed at ventilatory threshold, which was used to estimate a steady-state pace for the camber protocol, in conversation with the participant. Once warmed up, participants were instructed to wear a mouthpiece, nose clip, and heart rate monitor (POLAR Team Bluetooth Heart Rate Monitor, Bethpage, NY). The mouthpiece was attached to a metabolic cart (Vista MX, Vacumed, Ventura, CA) where metabolic data was recorded. The metabolic cart was calibrated according to manufacturer specifications, using known gas concentrations of 16.00% oxygen and 4.00% carbon dioxide. The metabolic measures were the following: VO_2_ (volume of oxygen consumption), V_E_ (expired ventilation), and RER (respiratory exchange ratio). These measures were recorded every 15 s using open circuit calorimetry, with peak oxygen consumption (VO_2peak_) recorded as the highest VO_2_ recorded in a 15 s period.

Participants began the GXT on the treadmill set at a 1% grade (TRACKMASTER, Jas Fitness System, Newton, KS). The GXT protocol began at 11.3 or 12.9 km/hr and treadmill speed was increased by 0.16 km/hr every 15 s until the participant reached volitional exhaustion, where they were then instructed to straddle the treadmill by suspending themselves from the handles and placing their feet on the rails of the treadmill. The goal was to reach volitional exhaustion within 8–12 min. Starting speeds were modified based on one’s fitness/speed from a recent 5 k effort, recent Strava paces, or in conversation with the participant. Following the GXT, participants were instructed to rest for 15 min to allow for adequate recovery before the camber running protocol.

During the 15 min rest period, the researchers analyzed the data to determine an appropriate pace for the next portion of the study as well as attach surface EMG and electromagnetic kinematic trackers to the necessary muscles and bony landmarks. To determine ventilatory threshold, the ventilatory equivalent method, or speed corresponding to a systematic increase in the ventilatory equivalent of oxygen (VE/VO_2_) without a concomitant increase in the ventilatory equivalent of carbon dioxide (VE/VCO_2_), was used to determine the speed at ventilatory threshold (Wasserman and McIlroy 1964). Participants then were asked to run between 0.8 and 1.6 km/hr below ventilatory threshold (in consultation with the participants) to ensure that participants were running at steady-state and not undergoing the slow component of VO_2_ during the trial. The average running speed for the camber protocol for all participants was 3.86 ± 0.36 m/s (8.63 ± 0.81 mph).

#### Camber running protocol

This portion of the study consisted of three 5 min runs with 5 min rest breaks in between each set to reduce the effect of fatigue. Each condition was a different camber (0°, 3°, and 6°), which were selected because of their similarity to normal road cambers. The order of these conditions was randomized prior to the study using a random number generator to eliminate the possibility of an order effect, and the speed (determined previously) remained the same throughout each camber condition. Cambers were established during the 5 min rest breaks by adding or removing wooden boards underneath the right side of the treadmill to mimic running on the left side of the road against traffic.

#### Metabolic data collection

Running economy was expressed as the steady-state VO_2_ in L/min and as running economy in W/kg as described previously (Kipp et al. 2018; Péronnet and Massicotte 1991). Briefly, W/kg were calculated using the following formula: ((16.89*VO_2_ (L/sec)) + (4.84*VCO_2_ (L/sec)*1000)/Body mass. Metabolic measures (VO_2_, V_E_, and RER) were collected every 15 s during the final 2 min of each 5 min trial to allow for participants to reach steady state. A steady-state was verified by a respiratory exchange ratio of less than 1 in each condition (0.86 ± 0.03 for 0 deg, 0.86 ± 0.03 for 3 deg, 0.87 ± 0.04 for 6 deg) as well as a steady and unchanging heart rate and VO2 (L/min) (Haverty et al. 1988).

#### Kinematic data collection

Kinematic measures were recorded during the final 2 min of each 5 min trial using four electromagnetic markers at a sampling rate of 100 Hz (Northern Digital, Waterloo, Ontario, Canada). These markers were firmly attached close to the skin on each of these bony landmarks: C7 vertebrae, right greater trochanter (GT), right fibular head, and right lateral malleolus. The placement of these sensors was reinforced with athletic tape and pre-wrap. At the start of each test session, participants stood still for 10 s with feet shoulder width apart and knees fully extended. The mean position of markers during this 10 s period was subtracted in the kinematic metrics to remove any biases in marker placements.

The kinematic metrics were calculated as follows. Trunk tilt in the sagittal plane was calculated as the arctangent of the horizontal position of C7 in the sagittal plane minus the horizontal position of the GT, divided by the vertical distance between C7 and GT. Trunk tilt in the frontal plane was calculated with the same method. Knee angles were calculated in the sagittal plane as the angle formed by the GT, right fibular head and right lateral malleolus. Hip flexion was calculated in the sagittal plane as the angle formed by the C7, GT, and right fibular head. Foot position was measured as the position of the right lateral malleolus. Vertical oscillation was measured as the vertical displacement of the GT.

Using the angles above, we calculated the following kinematic variables as shown in Table 1 in the appendix.

#### Electromyography data collection

Five surface EMG sensors were attached to the right and left vastus medialis, right and left lateral gastrocnemius, and right tibialis anterior (Delsys Wireless EMG, Natick, MA, USA). The surface of the skin was swabbed with alcohol wipes prior to application, and the electrodes were placed in a bipolar electrode configuration on the belly of each muscle, which were reinforced with pre-wrap. EMG signals were recorded using monitoring electrodes with full-surface solid adhesive hydrogel with on-site amplification, which were high-pass filtered using fourth order zero-lag Butterworth filters and subsequently smoothed using a root-mean-square (RMS) filter (30-ms symmetrical moving window with successive 1-ms steps). EMG signal amplitudes were normalized to the 0-degree condition, or control condition, for each subject, similar to that of Yaserifar et. al (Yaserifar and Souza Oliveira 2022), and to better understand how camber modifies electrical activity of different muscle groups. Five complete gait cycles per surface condition were analyzed for each subject for each muscle group, 4 min into each 5 min run. Each gait cycle was visually inspected to ensure that it was representative of a normal EMG pattern for each participant. In cases where EMG sensors slid down or fell off the leg completely, the data for the respective subject was either removed from analysis or adjusted to a different time point close to the 4 min mark when the sensor was still in its initial position. Four subjects were removed from analysis due to sensor issues.

#### Perceptual measures

RPE was recorded every 30 s during the GXT using the Borg 6–20 RPE scale (Borg, 1982). RPE was explained as the answer to the question “How hard do you feel like you are working?” (VanHaitsma 2023). When gathering RPE data, participants were asked to point to the corresponding number on a sheet of paper taped to the front of the treadmill. During the camber running protocol, RPE was recorded at the 3 min and 5 min marks (immediately before and after the 2 min kinematic data collection) to control for kinematic changes that may have occurred due to pointing.

### Statistics

A three-way (camber) repeated-measures ANOVA was conducted for all metabolic, kinematic, and EMG measures to evaluate camber main effects as well as group effects based on running economy differences using SPSS (Version 28). In instances where sphericity was violated, a Greenhouse–Geisser correction was used to adjust the degrees of freedom. If camber effects were significant, tests of within-subjects contrasts were used to determine which points differed from baseline. Significant camber effects and camber by group interactions were followed up with post-hoc pairwise comparisons. All data were presented as means and standard deviations, with significance set at α < 0.05.

## Results

### Metabolic measures

**Fig. 1:**
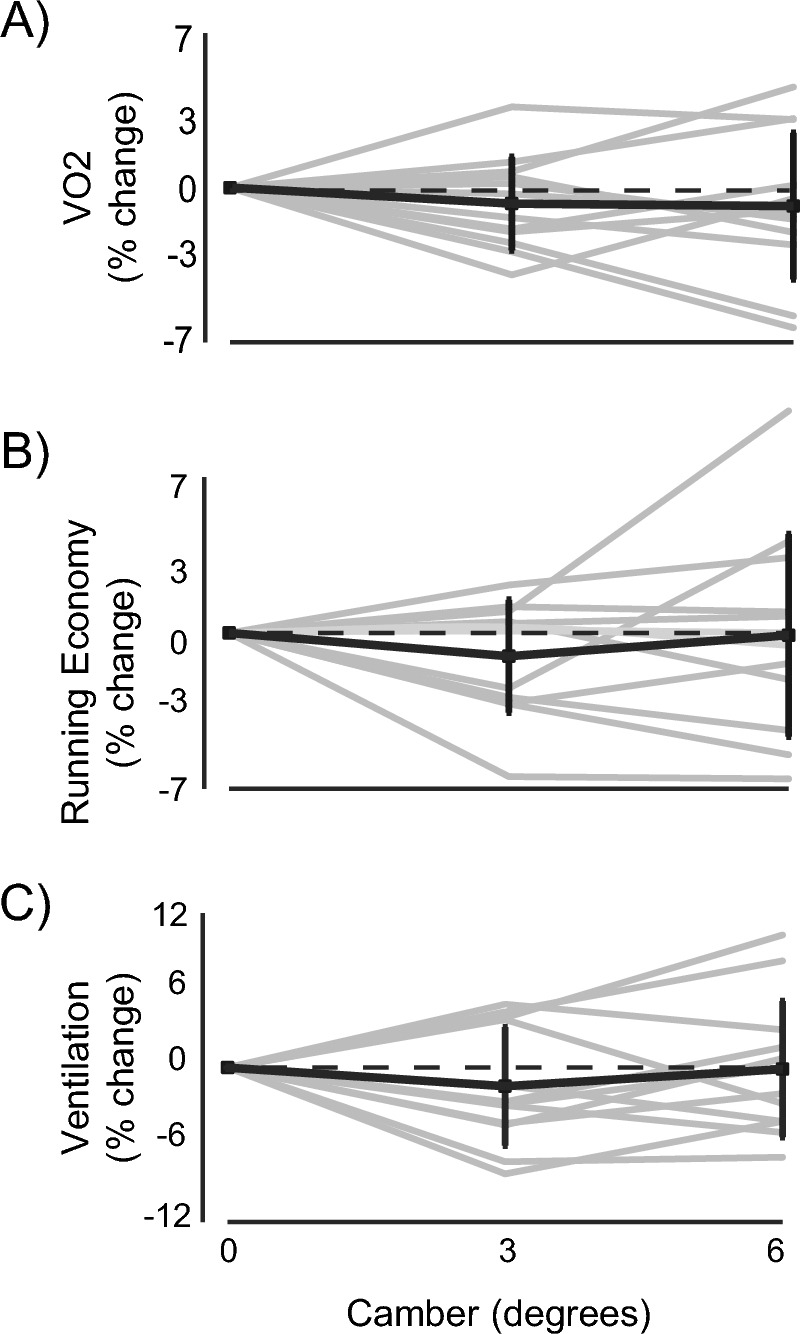
Metabolic changes due to changes in camber. The black dots represents the mean and standard deviation for each camber, the dashed grey lines represent individual data, the dashed black line on the percent change graphs represents no change from baseline. **A** Percent change in VO2 normalized to the 0 degree camber for the 3 and 6 degree camber, **B** Percent change in energetic cost normalized to the 0 degree camber for the 3 and 6 degree camber**, C** Percent change for ventilation in L/min normalized to the 0 degree camber for the 3 and 6 degree camber

Initial analyses revealed no camber effects for the metabolic data including VO_2_ L/min, VO_2_ mL/kg/min_,_ energetic cost (Fig. 1**)**, V_E_, HR, RER, and RPE (Table 1).

**Table 1:** Whole group metabolic variables with changes in camber

Camber	0 deg	3 deg	6 deg
VO_2_ (L/min)	3.59 ± 0.40	3.53 ± 0.39	3.53 ± 0.41
Energetic cost (W/kg)	3.51 ± 0.43	3.47 ± 0.44	3.50 ± 0.46
V_E_ (L/min)	80.95 ± 8.8	79.70 ± 8.7	80.77 ± 9.1
HR (bpm)	168.10 ± 15.1	168.55 ± 13.1	168.46 ± 13.9
RER	0.86 ± 0.03	0.86 ± 0.03	0.87 ± 0.04
RPE (Borg scale)	13.78 ± 1.6	13.42 ± 1.8	13.92 ± 1.8

As a whole, there were no significant changes in metabolic parameters

### Kinematic measures

#### Trunk

**Fig. 2:**
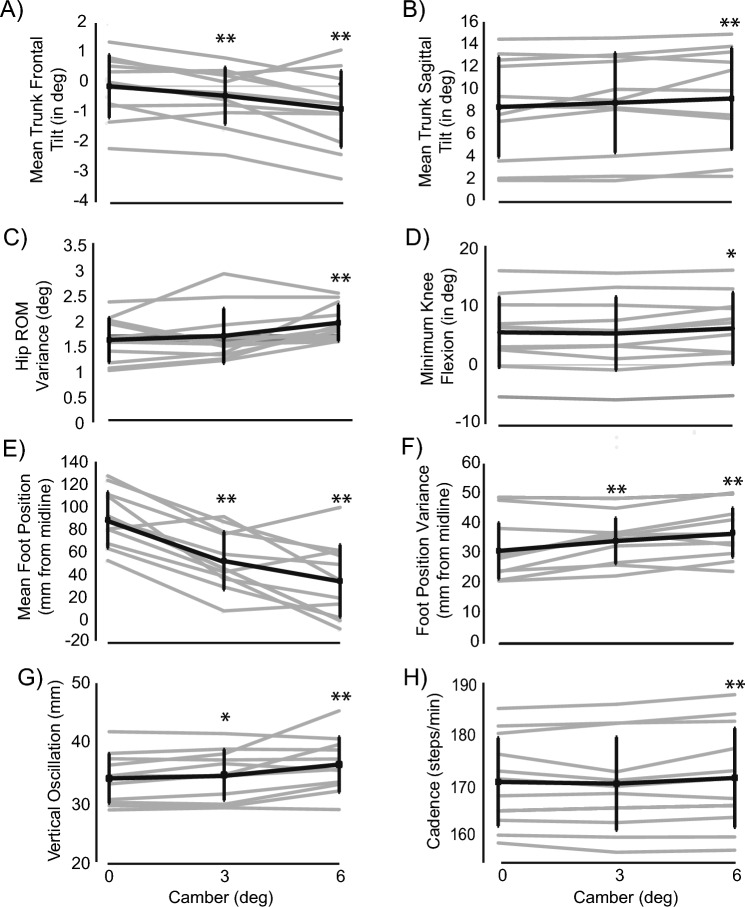
Kinematic changes due to changes in camber. The black dots represents the mean and standard deviation for each camber, the dashed grey lines represent individual data, the dashed black line on the percent change graphs represents no change from baseline. **A** Mean changes to trunk tilt in the frontal plane, **B** Mean changes to trunk tilt in the sagittal plane, **C** Mean changes in the range of motion at the hip, **D** Mean changes in the minimum flexion at the knee, **E** Changes in the mean foot position at landing from the midline, **F** Changes in the variance of the foot position at landing, **G** Changes in vertical oscillation, **H** Changes in cadence

Camber main effects were found for average trunk tilt/lean angles in both the sagittal and frontal planes. Runners tended to tilt their trunk more downslope (towards the lower end of the treadmill) with increasing camber (*F*(1.261,11.352) = 7.692, p = 0.014), as shown in Fig. 2A. Tests of within-subjects contrasts were performed to further examine this effect. Runners tilted on average 0.30 ± 0.09 deg more downhill from the baseline trial to the 3 deg camber (*F*(1,9) = 10.673, p = 0.01) and 0.73 ± 0.2 deg from the baseline trial to the 6 degree camber (*F*(1,9) = 10.666, p = 0.01).

Runners also tended to lean forward more with increasing camber (*F*(2,18) = 3.405, p = 0.056), as shown in Fig. 2B a preplanned contrast comparing sagittal lean found that runners leaned on average 0.71 ± 0.3 deg more downhill at 6 deg than at baseline (*F*(1,9) = 5.486, p = 0.033), but there were no significant differences at 3 deg (p > 0.05).

#### Knee and hip

Main effects of camber were found for both minimum knee flexion and hip range of motion variance (Table 2 in the appendix). Runners had lower minimum knee flexion angles (that is, they had greater knee flexion in stance) with increasing camber (*F*(2,18) = 4.008, p = 0.036), as shown in Fig. 2D. Pairwise comparisons showed a significant decrease of minimum knee flexion from 3 deg (5.28 ± 6.2 degrees) to 6 deg camber (6.13 ± 6.0 degrees; p = 0.038).

Hip range of motion variance in the sagittal plane varied to a greater degree with increasing camber (*F*(2,18) = 3.957, p = 0.038), Fig. 2C. Tests of within-subjects contrasts displayed a significantly greater degree of variance (0.326 ± 0.1 deg) in their hip joint range of motion at 6 deg compared to baseline (*F*(1,9) = 5.931, p = 0.038). Hip range of motion variance between 3 and 6 deg did not reach statistical significance (p = 0.059).

Hip vertical oscillation also had a significant camber effect (F(2,18) = 5.117, p = 0.017), Fig. 2G. Tests of within-subjects contrasts demonstrated an increased vertical oscillation while running on a 6 deg camber (36.7 ± 4.65 mm) as compared to level ground (34.4 ± 4.2 mm; F(1,9) = 5.683, p = 0.041) though there was no significant difference for a 3 deg camber (35.0 ± 4.3 mm) as compared to running on level ground (F(1,9) = 4.189, p = 0.071).

#### Cadence and foot positioning

Camber main effects were found for average foot position and foot position variance (Table 2 in the appendix). Runners tended to run with their right foot more downslope with increasing camber (*F*(2.20) = 29.837, p < 0.001). The right foot was 36.33 ± 5.8 mm closer to midline at 3 deg (*F*(1,10) = 39.239, p < 0.001) and 54.23 ± 7.8 mm closer to midline at 6 deg (*F*(1,10) = 47,823, p < 0.001) (Fig. 2E). When examining foot position only at landing (in stance phase), similar trends were found. Subjects landed with their right foot closer to mediolateral midline as camber increased (*F*(2,20) = 25.934, p < 0.001), with their right foot landing 37.23 ± 6.4 mm closer to midline at 3 deg (*F*(1,10) = 33.719), p < 0.001) and 53.96 ± 8.2 mm closer to midline at 6 deg (*F*(1,10) = 43.089, p < 0.001).

Runners had greater variance in foot position with increasing camber (*F*(2,9) = 14.009, p < 0.001), with foot position varying 3.31 ± 1.1 mm more at 3 deg (*F*(1,10) = 8.235, p = 0.016) and 6.06 ± 1.1 mm more at 6 deg (*F*(1,10) = 29.485, p < 0.001) (Fig. 2F). When adjusted for foot position at landing, similar trends were found. Runners had greater variance with increasing camber (*F*(2,9) = 7.473, p = 0.004), with landing foot position varying 3.01 ± 1.3 mm more at 3 deg (*F*(1,10) = 8.235, p = 0.016) and 5.39 ± 1.4 mm more at 6 deg (*F*(1,10) = 15.589, p = 0.003).

There was a significant camber effect on cadence (*F*(2,20) = 4.550, p = 0.024), Fig. 2H**.** Tests of within-subjects contrasts showed that runners as a whole increased cadence 0.81 ± 0.3 steps/min from baseline to 6 deg (*F*(1,10) = 6.060, p = 0.034).

### Electromyography

No main effects of camber were found for EMG measures on the right or left vastus medialis or the tibialis anterior.

**Fig. 3:**
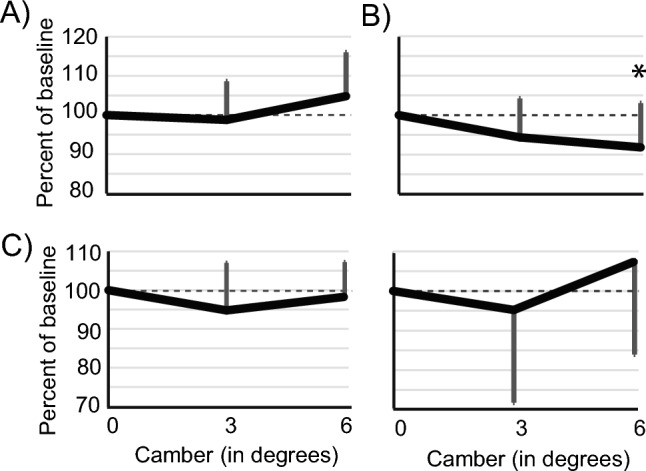
Electromyography area under the curve data across camber. Data are presented as mean ± SD. **A** Left gastrocemius lateralis, **B** Right gastrocnemius lateralis, **C** Left vastus medialis, **D** Right vastus medialis

For the left lateral gastrocnemius (LGL), there were no camber effects for EMG duration, EMG total activity (area under the curve), or EMG peak amplitude (Fig. 3), though there was a trend towards a camber effect for area under the curve (F(2,9) = 3.118, p = 0.094) with the largest increases at 6 deg (4.9 ± 19.8%).

For the right lateral gastrocnemius (RGL), however, there were significant effects for each of the three measures. EMG duration had no camber main effect, but there was a significant camber effect for area under the curve (*F*(2,18) = 3.832, p = 0.041), with AUC decreasing in the uphill leg as camber increased (Fig. 3**)**. At 3 deg, EMG total activity had a non-significant decrease of 5.49% ± 9.9% (*F*(1,9) = 4.518, p = 0.062). At 6 deg, EMG total activity decreased by 8.05% ± 11.3% (*F*(1,9) = 5.611, p = 0.042).

EMG peak amplitude also showed a significant camber effect (*F*(2,18) 6.059, p = 0.010), with an overall decrease in EMG peak amplitude in the RGL as camber increased. At 3 deg, EMG peak amplitude decreased by 8.60% ± 7.4% (*F*(1,9) = 13.596, p = 0.005). At 6 deg, EMG peak amplitude decreased by 8.07% ± 8.2% (*F*(1,9) = 8.578, p = 0.017).

## Discussion

### Overall findings

The current study investigated the effects of running on cambered surfaces of 3 and 6 degrees on running economy and biomechanics. By analyzing its effects on metabolic, kinematic, and EMG patterns at a steady-state pace slightly below ventilatory threshold on a group of trained runners, several trends were found. As hypothesized, participants experienced no significant decrements in running economy (expressed as either VO_2_ in L/min or as W/kg) despite experiencing significant changes in kinematic and EMG patterns. These findings are consistent with previous literature which also found no metabolic decrements in cambered running of up to 6 deg despite significant changes in gait characteristics (Elmer and Asbill 2023). Our study expands on this previous literature in two important ways. First, we examined additional kinematic variables that provide more detail into running adaptations on a camber surface. Second, by using bilateral EMG measures, we were able to detect an asymmetry in the gastrocnemius muscle. These two topics are expanded in the discussion below.

### Kinematic adaptations of the leg and feet

The most robust camber effects were found in the lower extremity. Effect sizes ranged from about 7.5 to 43 in the foot and 4.5 to 6 in cadence, while effect sizes for the knee, hip, and trunk were generally lower (3.2 to 6.2 in the knee, 4 to 6 in the hip, and 3.4 to 10.6 in the trunk). Increasing camber was associated with higher cadence, greater variance in foot placement, and a shift in foot position toward the downhill side. The increase in cadence and foot placement variance could be related to balance as people are known to increase cadence (Rogers et al. 2008) and variance in foot placement to maintain balance (Kuo and Donelan 2010). We can speculate two possible causes of the downhill shift in foot position. First, the runners may have had a tendency to push off perpendicular to the surface, which would have actually sent them slightly downhill. Second, we noted a small average body tilt downhill with larger camber. The gravitational force on a tilted body tends to further push the body downhill (Peterka 2002). Together, these findings deviate from straight running on a flat surface (Arellano and Kram 2011).

The finding that changes in camber affected the foot and ankle more than superior body segments suggests that individuals adopt a bottom-up adaptation approach. For example, the subtalar joint is known to stabilize the ankle and foot during running, and when the camber exceeds the capabilities of the subtalar joint, the task of attenuating camber has been shown to get transferred to the knee joint (Gow 2005; Quintana 2020; Gehlsen et al. 1989; Elmer and Asbill 2023; Sussman et al. 2001; Willwacher et al. 2013; Unfried et al. 2013). These bottom-up changes appear to be a reflection of the body’s attempt at reversing an environmental leg-length inequality induced by camber. Anatomical leg length inequalities have been shown to alter the pattern of mechanical stress within a joint, concurrent with the pelvis tilting toward the long leg to create a functional scoliosis (Mccaw 1992).

The current study also found that the upslope knee went into greater flexion to functionally shorten the ipsilateral limb. Consistent with these results, previous work has shown greater mean knee flexion at initial contact and toe off for the upslope leg at 2.5 and 5.0 deg of camber at 7 mph (Sussman et al. 2001). Consistent with the bottom-up process of adaptation, the same study found no significant changes in the hip joint, although the range of motion of the hip in our study did vary with camber. Finally, we found a significant increase in vertical oscillation at the hip with increasing camber. The increase may point to an interaction with the knee, where more flexion of the upslope knee during the stance phase may lead to ipsilateral hip drop (and overall greater vertical oscillation in the gait cycle), and could increase the energetic cost of running in some cases (Folland et al. 2017).

### Kinematic adaptations of the trunk

The current study is the first to examine trunk tilt while running on a camber. Our results are consistent with existing literature that has documented typical frontal plane balance responses to tilted surfaces (Goodworth and Peterka 2009, 2012). In particular, the natural response is to lean toward the tilted surface (ie, if the surface tilts downhill to the left, the person tends to lean their body to the left). The origin of this lean is typically attributed to an intrinsic stiffness mechanism that orients the body toward the surface and to a sensorimotor integration mechanism where somatosensory feedback orients to the surface and visual and vestibular orient the body upright (Goodworth and Peterka 2009; Peterka 2012). The relatively small trunk leans suggest that the lower body adaptations were largely effective in adjusting to the cambered surface so that experienced runners did not need to exhibit large trunk leans. Previous cambered running studies suggested that maintaining an upright trunk is a primary goal of lower limb adaptations (Damavandi et al. 2016; Gehlsen et al. 1989; Elmer and Asbill 2023; Unfried et al. 2013).

We did not find any significant changes in trunk tilt variance in either plane. One explanation may be that our experienced runners were already adept at stabilizing their trunk motion, as previous research found that maintaining a steady trunk posture in the frontal and sagittal planes during running was related to a better running economy (Folland et al. 2017). Finally, we note that the lower body likely also adapted with a downhill tilt (Goodworth and Peterka 2012). However, to accurately measure this in the current study, we would have needed kinematic sensors on both feet to capture the stance phase (which was not possible with our equipment).

One practical implication of our kinematic finding is related to injury. Asymmetries in loading are sometimes associated with heightened risk of injury from unilateral overuse. Muscle activation asymmetries have been reported in low back pain (Oddsson and Luca 2003; Dieën et al. 2003). In the current study, the statistically significant trunk kinematic changes were small, but previous research comparing pathological groups to controls also found small significant kinematic differences (1 to 3 degrees) with similar effect sizes as the current study (Chen et al. 2024; Sung and Danial 2018). These results suggest that small kinematic different could still be meaningful, and we anticipate other runners with less experience would be more affected by camber.

Another practical scenario is where a cambered surface is sometimes paired with curved paths (such as “banking” on a track). This scenario is more complex than the current treadmill study because running along a curved path introduces additional centripetal accelerations, such that faster speeds and smaller radii are associated with larger centripetal accelerations and often larger inward leans (Goodworth et al. 2013). Thus, while our current study examined trunk tilt in response to camber, in many real life running scenarios, tilt would be affected by both camber + banked scenarios, as documented in the lower extremity in previous running studies (Garie 2006; Gow 2005; Quintana 2020).

### EMG of the lower leg

Consistent with the bottom-up adaptation concept, we found significant effects of camber on EMG in the lower leg right gastrocnemius muscle and not the more superior muscles that were measured in this study (eg, vastus medialis). The EMG of the lateral gastrocnemius in the current study reflects this: while camber did not significantly alter the lateral gastrocnemius activity of the downslope leg, there were still EMG trends that were opposite compared to the upslope right leg. These trends loosely align with the body’s attempt to functionally elongate the downslope leg while shortening the upslope leg to better attenuate the asymmetrical loading patterns induced by camber (White et al. 2004). Among runners with a simulated leg length inequality (achieved by elevating the sole of one shoe by 1.31 cm), the shorter (downslope) limb sustained greater loads and loading rates (White et al. 2004). In the current study, the lateral gastrocnemius EMG of the downslope leg trended towards increasing with camber while that of the upslope leg significantly decreased. This decrease in right gastrocnemius EMG may point to the upslope limb taking relatively smaller loads and loading rates with increasing camber with the downslope limb taking larger loads (White et al. 2004). This asymmetrical muscle activation pattern, with an increased loading of the downslope limb, may cause altered fatigue characteristics leading to earlier muscle fatigue (Boyas and Guével 2011) or may contribute to the 18–70% of marathoners that experience exercise-associated muscle cramp (EAMC) (Maughan and Shirreffs 2019). Increased neuromuscular fatigue may precede EAMC, particularly when athletes race faster than their training predicts (Drew 2006).

Other studies have reported different EMG patterns. One study, using a similar protocol, found some of the same kinematic adaptations in the lower leg, but also found significant differences with camber in the contralateral muscle groups as lateral gastrocnemius activation was increased on the downhill leg and increased on the uphill leg (Unfried et al. 2013). Another did not find any significant differences with the same muscle groups despite finding similar kinematic changes and having similar cambers, though the gastrocnemius location was not specified (lateral vs medial head) (Elmer and Asbill 2023). Thus, the state of changes in camber on muscle activation is still being developed, and our study clarifies the existing knowledge.

### Limitations

The greatest limitation to this study was the small sample size. From a sample size of 15 subjects, data from 3 runners had to be discarded due to errors in the data and equipment. Several statistical tests in our results had p-values between 0.05 and 0.1. A larger sample size with similar effect sizes and variance would have greater power to detect changes and might result in p-values below 0.05.

In addition to the small sample size, this study did not recruit women due to the shoes that were available for the runners. These two shoe sizes were sufficient for the participants of this study, though it should be noted that sometimes the shoes were about a half-size different from an individual’s typical shoe size, which could potentially alter the kinematics of the participants. Additionally, by having participants run in shoes to which they are not accustomed, there are further potential changes to gait characteristics and therefore kinematics. However, because participants are experienced runners, the participants have run in a wide variety of shoes during the course of their running career, and they are able to quickly adjust to the shoe.

The protocol itself also introduced a limitation, as we elected to do a maximal GXT prior to the cambered trials. This does raise the possibility of introducing a fatiguing element to the trials; however, participants were all well-trained. Additionally, future studies could examine the initial transition steps to the introduction of a cambered surface since these could vary from the steady state analysis in our current study. Additionally, we only completed one trial on each camber, contrary to current best practices.

The current equipment for collecting kinematic and EMG data were sufficient, however, the lack of force plates and video equipment in the methodology made it difficult to differentiate kinematic and EMG differences between stance/swing phases of running.

We also did not screen for leg length inequality, which could have explained differences between individuals. Having information on existing leg length inequality may have provided a clearer picture as to the variability in kinematic adaptations that were occurring for different individuals and if the adaptations were due to the camber and/or a potential individual leg length inequality.

## Conclusions/implications

The findings of this study have important implications on the running community, particularly for those athletes who are looking for their best performances while running on the roads. The results suggest that running on a camber of up to 6 deg does not cause decrements to running economy. Although significant kinematic and EMG adaptations occurred, they appear to occur within a bottom-up adaptation that did not incur metabolic costs. Runners, therefore, do not need to stay on the crown/flat part of the road to avoid metabolic decrements. Many of the kinematic and EMG changes were significant but small in response to camber and calls for further research on possible implications of these asymmetries in regards to injury or fatigue during training and long duration events.

## Supplementary Information

Below is the link to the electronic supplementary material.

Supplementary file1 (DOCX 19 KB)

## Data Availability

Data will be made available on reasonable request.
