# Supplementary material for: Does running at an angle affect running economy? Metabolic, kinematic, and EMG adaptations to running on a camber
Source: Eur J Appl Physiol. 2025 Oct 29;126(4):2097–107. doi: 10.1007/s00421-025-06034-3 (PMC13171681; doi:10.1007/s00421-025-06034-3)
Supplement: Supplementary file 1 — Supplementary file1 (DOCX 19 KB) [file 421_2025_6034_MOESM1_ESM.docx]

Table 1. A list of all the kinematic variables and the basis by which they were calculated.

| **TRUNK** | |
| --- | --- |
| Mean trunk tilt in frontal plane (deg) | Mean of trunk tilt across entire trial, which quantifies right uphill (+) or left downhill (-) bias in trunk lean with respect to vertical |
| Trunk tilt variance in frontal plane (deg) | Standard deviation of trunk tilt angle |
| Mean trunk tilt in sagittal plane (deg) | Mean of trunk tilt across entire trial, which quantifies forward (+) or backward (-) bias in trunk lean with respect to vertical |
| Trunk tilt variance in sagittal plane (deg) | Standard deviation of trunk tilt angle |
| **KNEE** | |
| Minimum knee flexion (deg) | Minimum of knee angle of the average gait cycle, where 0 indicates a straight knee (full extension) |
| Maximum knee flexion (deg) | Maximum knee flexion angle of the average gait cycle, where larger values indicate more flexion (in swing phase) |
| Knee range of motion (deg) | Max-min of the knee angle of the average gait cycle |
| Knee range of motion variance (deg) | Standard deviation across gait cycles of the max-min knee angle |
| **HIP** | |
| Minimum hip flexion (deg) | Smallest hip flexion angle of average gait cycle, where negative values indicate hip extension |
| Maximum hip flexion (deg) | Largest hip flexion angle of average gait cycle, larger positive values indicate more hip flexion |
| Hip range of motion (sagittal plane) (deg) | Max-min of hip flexion angle of the average gait cycle |
| Hip range of motion variance (deg) | Standard deviation across gait cycles of the max-min hip angle |
| Vertical oscillation (mm) | Standard deviation of greater trochanter in the vertical direction |
| **FEET** | |
| Mean foot position (mm) | Mean foot position measured at malleolus across the entire trial, where 0 is midline |
| Foot position variance (mm) | Standard deviation of foot position across the entire trial |
| Mean foot position at landing (mm) | Foot position in stance phase averaged across cycles, where 0 is midline |
| Foot position variance at landing (mm) | Standard deviation across gait cycles of foot position in stance phase |
| Cadence (step/min) | Number of steps divided by the time the steps occurs for entire trial |

**Table 2** Descriptive values for kinematic parameters during running at 0 deg, 3 deg, and 6 deg. Values are expressed in mean ± SD. In anteroposterior (sagittal plane), positive values represent forward/anterior movement. In mediolateral (frontal plane), positive values represent movement to the right (ie, uphill). For joint angles, the values represent absolute flexion (degree of flexion from full extension).

| Camber | 0 deg | 3 deg | 6 deg | Significance |
| --- | --- | --- | --- | --- |
| Mean trunk tilt in frontal plane (deg) | 0 ± 1.0 | -0.32 ± 0.9 | -0.76 ± 1.3 | ** |
| Trunk tilt variance in frontal plane (deg) | 2.07 ± 0.6 | 2.05 ± 0.6 | 2.03 ± 0.5 |  |
| Mean trunk tilt in sagittal plane (deg) | 8.42 ± 4.4 | 8.80 ± 4.4 | 9.17 ± 4.5 | ** |
| Trunk tilt variance in sagittal plane (deg) | 2.04 ± 0.4 | 1.98 ± 0.4 | 2.02 ± 0.5 |  |
| Minimum knee flexion (deg) | 5.48 ± 5.9 | 5.28 ± 6.2 | 6.13 ± 6.0 | ** |
| Maximum knee flexion (deg) | 91.07 ± 6.8 | 92.94 ± 7.9 | 93.27 ± 8.6 |  |
| Knee range of motion (deg) | 85.60 ± 7.7 | 87.66 ± 8.9 | 87.14 ± 9.6 |  |
| Knee range of motion variance (deg) | 2.95 ± 1.0 | 2.89 ± 0.7 | 2.99 ± 0.8 |  |
| Minimum hip flexion (deg) | -10.55 ± 3.9 | -10.53 ± 3.9 | -10.54 ± 3.9 |  |
| Maximum hip flexion (deg) | 20.52 ± 4.9 | 20.82 ± 4.7 | 21.49 ± 6.3 |  |
| Hip range of motion (sagittal plane) (deg) | 35.49 ± 8.0 | 35.76 ± 8.1 | 35.02 ± 6.7 |  |
| Hip range of motion variance (deg) | 1.57 ± 0.4 | 1.66 ± 0.5 | 1.92 ± 0.4 | ** |
| Hip vertical oscillation (mm) | 34.38 ± 4.2 | 34.97 ± 4.3 | 36.70 ± 4.6 | ** |
| Mean foot position (mm) | 89.28 ± 24.9 | 52.95 ± 25.8 | 35.04 ± 32.07 | ** |
| Foot position variance (mm) | 30.98 ± 9.37 | 34.29 ± 7.6 | 37.04 ± 8.2 | ** |
| Mean foot position at landing (mm) | 78.12 ± 27.7 | 40.89 ± 27.0 | 24.16 ± 36.0 | ** |
| Foot position variance at landing (mm) | 20.52 ± 4.2 | 23.53 ± 4.8 | 25.9 ± 5.5 | ** |
| Cadence (step/min) | 170.90 ± 8.6 | 170.57 ± 9.1 | 171.71 ± 9.7 | ** |

**p < .05 for a camber main effect.
